# Association between Carotid Intima‐Media Thickness and White Matter Hyperintensity Volume

**DOI:** 10.1002/alz70856_104328

**Published:** 2025-12-26

**Authors:** Ambar Kulshreshtha, Alvaro Alonso, Yantong Kelly, Deqiang Qiu, Joe Brown, Arshed Quyyumi, James J. Lah

**Affiliations:** ^1^ Emory University School of Medicine, Atlanta, GA, USA; ^2^ Emory University Rollins School of Public Health, Atlanta, GA, USA; ^3^ Emory University School of Medicine, ATLANTA, GA, USA; ^4^ Emory Goizueta Alzheimer's Disease Research Center, Atlanta, GA, USA; ^5^ Emory University, ATLANTA, GA, USA; ^6^ Goizueta Alzheimer's Disease Research Center, Emory University, Atlanta, GA, USA

## Abstract

**Background:**

White matter hyperintensity (WMH) volume is a recognized marker of small vessel disease associated with cognitive decline. Carotid Intima‐Media thickness (CIMT), an established non‐invasive measure of early atherosclerosis, provides insights into vascular contributions to brain aging and neurodegenerative conditions like dementia.

**Methods:**

We analyzed data from 872 cognitively normal participants in the ongoing Emory Healthy Brain Study (EHBS) in Atlanta, GA. CIMT was measured following an established protocol, and WMH volume was quantified from 3.0 Tesla MRI images. A linear mixed model was employed to examine the association between CIMT and WMH volume, adjusting for covariates such as age, gender, education level, and history of hypertension and diabetes. To explore effects in different groups, we stratified analyses by age, race, and sex.

**Results:**

The mean age of participants was 63.3 years (SD = 6.5), with 66% female, 94% White, and 6% African American. The mean educational attainment was 17 years. The mean carotid maximum was 0.80 mm (SD = 0.14), and the mean WMH volume was 7.69 cm3 (SD = 18.6). Age, history of hypertension, and diabetes were key predictors of CIMT. In the unadjusted analyses, CIMT was positively associated with WMH volume (β = 2.81 per 1‐SD increment in CIMT, 95% CI [1.51, 4.11], *p* < 0.01). After adjusting for demographics and CVD risk factors, CIMT remained significantly associated with WMH volume (β = 2.38 per 1‐SD increment, 95% CI [0.01, 4.75], *p* =  0.048). Stratified analyses revealed that in individuals aged <60 years, CIMT was more strongly associated with WMH volume (β = 5.19, 95% CI [1.71, 8.67]) compared to those aged ≥60 years (β = 1.42, 95% CI [‐1.69, 4.54]). Similarly, the association was stronger in women (β = 3.03, 95% CI [1.11, 4.96], *p* < 0.01) than in men (β = 1.80, 95% CI [‐2.77, 6.37], *p* =  0.43).

**Conclusion:**

Our findings suggest an independent association between CIMT and WMH volume, with stronger effects observed in women and individuals under 60. Thickened carotid arteries can indicate systemic vascular pathology that could affect smaller blood vessels in the brain, leading to WMH and potentially leading to cognitive decline.

